# The safety and efficacy of systemic delivery of a new liver-de-targeted TGFβ signaling inhibiting adenovirus in an immunocompetent triple negative mouse mammary tumor model

**DOI:** 10.1038/s41417-024-00735-1

**Published:** 2024-01-24

**Authors:** Soon Cheon Shin, Renee E. Vickman, Beniamin Filimon, Yuefeng Yang, Zebin Hu, Kathy A. Mangold, Bellur S. Prabhakar, Hans Schreiber, Weidong Xu

**Affiliations:** 1 Cancer Gene Therapy Program, Department of Medicine, NorthShore University HealthSystem, an Academic Affiliate of the University of Chicago Pritzker School of Medicine, Endeavor Health Medical Group, Evanston, IL USA; 2 Center for Personalized Cancer Care, Department of Surgery, NorthShore University HealthSystem, an Academic Affiliate of the University of Chicago Pritzker School of Medicine, Endeavor Health Medical Group, Evanston, IL USA; 3 Department of Experimental Medical Science and Key Laboratory of Diagnosis and Treatment of Digestive System Tumors of Zhejiang Province, Ningbo, China; 4 National Institutes for Food and Drug Control, Beijing, China; 5 Department of Pathology and Laboratory Medicine, NorthShore University HealthSystem, Endeavor Health Medical Group, Evanston, IL USA; 6 Department of Pathology, The University of Chicago, Chicago, IL USA; 7 Department of Microbiology and Immunology, University of Illinois College of Medicine, Chicago, IL USA

**Keywords:** Breast cancer, Cancer immunotherapy

## Abstract

Aberrant TGFβ signaling is linked to metastasis and tumor immune escape of many cancers including metastatic triple negative breast cancer (mTNBC). Previously, we have found that oncolytic adenoviruses expressing a TGFβ signaling inhibitory protein (sTGFβRIIFc) induced immune activation in a mouse TNBC (4T1) immunocompetent subcutaneous model with intratumoral injection. Systemic administration of adenoviruses can be a superior route to treat mTNBC but faces the challenges of increased toxicity and viral clearance. Thus, we created a liver-de-targeted sTGFβRIIFc- and LyP-1 peptide-expressing adenovirus (mHAdLyp.sT) with enhanced breast cancer cell tropism. Its safety and immune response features were profiled in the 4T1 model. Our data showed that the systemic administration of mHAdLyp.sT resulted in reduced hepatic and systemic toxicity. mHAdLyp.sT was also effective in increasing Th1 cytokines and anti-tumor cell populations by cytokine analysis, spleen/tumor qRT-PCR, and flow cytometry. We further tested the therapeutic effects of mHAdLyp.sT alone and in combination with immune checkpoint inhibitors (ICIs). mHAdLyp.sT alone and with all ICI combinations elicited significant inhibition of lung metastasis by histological analysis. When mHAdLyp.sT was combined with both anti-PD-1 and anti-CTLA-4 antibodies, primary 4T1 tumor growth was also significantly inhibited. We are confident in advancing this new treatment option for mTNBC.

## Introduction

Triple-negative breast cancer (TNBC) is a highly heterogeneous disease and often associated with a poor prognosis, particularly for patients diagnosed with late-stage metastatic cancers. Recent advances in immunotherapy with the development of immune checkpoint inhibitors (ICIs), have changed the treatment paradigm of TNBC, with new FDA approvals and many new clinical trials in progress [1–3]. Although current TNBC immunotherapy strategies are effective in early stage TNBC patients, only modest clinical responses are observed in patients with metastatic disease. This may be because late-stage tumors are often highly immunosuppressive, thus reestablishing a favorable immune environment; therefore, more effective and long-lasting anti-cancer responses with combination therapies is essential [3]. In recent years, many studies have shown that TGFβ signaling plays a central role in tumor immune evasion and resistance to ICIs [4, 5]. We have previously shown that tumor stromal expression of TGFβ-1 is associated with TNBC and is a poor prognostic marker of overall survival in breast cancer patients, which is consistent with other studies that linked TGFβ signaling to drug resistance and poor survival rate in TNBC patients [6–8]. Therapeutic strategies to block TGFβ signaling in advanced cancers by inhibitory antibodies or fusion proteins have been the focus of several clinical investigations, and some of them involve patients with metastatic TNBC (mTNBC) [9–11]. Thus, investigations combining TGFβ pathway inhibitors with ICIs holds promise for the treatment of mTNBC.

We previously reported that TGFβ blockade by direct inoculation of an oncolytic adenovirus expressing a fusion protein with soluble TGFβ receptor II and the human IgG Fc fragment (sTGFβRIIFc) into subcutaneous mouse TNBC (4T1) tumors can inhibit protumorigenic signals and induce immune activation [12]. It can also enhance the antitumor responses of ICIs (anti-PD-1 and anti-CTLA-4 antibodies) in this immunocompetent mouse model. Although direct inoculation of adenovirus showed favorable results, the preferred route to deliver adenoviral vectors would be via systemic administration for metastatic cancers [13, 14]. Key challenges in the use of Ad5-based adenoviruses for systemic administration are increased liver/systemic toxicities, quick viral clearance, and limited tumor tropism. To limit hepatic and systemic toxicities, we previously created a liver de-targeted oncolytic adenovirus (mHAd.sT) expressing sTGFβRIIFc that has Ad5/48 chimeric hexon, which has a reduced capacity to bind with blood coagulation Factor X (FX) when administered intravenously [15, 16]. Because Ad5-FX complex is the major mechanism of hepatic sequestration, systemic toxicity, and viral clearance in mouse models [17, 18], we were able to use this adenovirus at the optimal dose to achieve better inhibitory effects on skeletal metastases of both human breast cancer and prostate cancer cells in immunodeficient bone metastasis mouse models [15, 16]. Recently, to enhance tumor tropism, we further engineered our sTGFβRIIFc expressing adenoviruses with a 9-amino acid-long tumor homing-cell penetrating peptide (LyP-1) into the HI loop of Ad5 adenoviral fiber to generate AdLyp.sT from Ad.sT, and mHAdLyp.sT from mHAd.sT [6]. Both AdLyp.sT and mHAdLyp.sT bind to LyP-1 receptor, which has been shown to be expressed on the breast cancer cell surface, tumor macrophages, and tumor lymphatics, but not readily detectable on normal tissues [19, 20].

The main goal of this study is to further test hepatic/systemic toxicity, tumor tropism, immunomodulatory factor expression, immune cell response, and the therapeutic efficacy of mHAdLyp.sT in the immunocompetent mouse TNBC (4T1) model. We use Ad.sT, AdLyp.sT, and mHAd.sT as the controls for our toxicity and immune mechanism studies. To evaluate mHAdLyp.sT’s effects on 4T1 tumor growth and metastasis we compare it with ICIs (anti-PD-1 and anti-CTLA-4 antibodies) and assess if it can synergize with ICIs to inhibit tumor growth and metastasis. We report here that intravenous delivery of mHAdLyp.sT has reduced hepatic uptake and hepatic/system toxicity but retains tumor tropism. mHAdLyp.sT is also potent in generating anti-tumor cytokine and immune cell responses, both systemically and in the tumor microenvironment (TME). It can enhance anti-tumor and anti-metastasis efficacy of anti-PD-1/anti-CTLA-4 therapy as well. Overall, our studies suggest that mHAdLyp.sT in combination with ICIs is suitable to be developed into clinical trials as a new systemic treatment option for mTNBCs, which is still particularly devastating to many patients.

## Materials and Methods

### Cell lines, adenoviruses, and antibodies

Mouse mammary tumor cell line, 4T1, was purchased from ATCC (Manassas, VA), and maintained in the lab as described previously [6, 12, 21]. ATCC STR profiling test for cell line authentication was recently performed with the result of 98% match of the database profile of ATCC 4T1 cell line (CRL-2539) (ATCC STR profiling test FTA Barcode: MUSA3575; Sales Order: SO2111801; Completed: 11/30/2023). The only difference is that our cell line doesn’t have allele 19 in STR marker 11-2, and may be the result of our attempt to subclone it in the past to have higher propensity to metastasize spontaneously to lung and bone. It was also retested recently and confirmed to be free of contamination of Ectromelia, EDIM, LCMV, LDEV, MHV, MNV, MPV, MVM, Mycoplasma pulmonis, Mycoplasma spp., Polyoma, PVM, REO3, Sendai, and TMEV by IMPACT III PCR Profile (IDEXX BioAnalytics, Columbia, MO, Case # 112172-2023, Completed: 5/3/2023). To create oncolytic adenoviruses viral or target genes were modified and cloned in a shuttle vector and subjected to homologous recombination with adenoviral genomic DNA derived from adenoviral mutant dl01/07 using published methods [6, 16, 22–24]. Ad.sT is the original cytomegalovirus (CMV) promoter-regulated Ad5-based virus expressing sTGFβRIIFc (named as Ad.sTβRFc in some of our previous publications) [22–24]. For hexon-chimeric adenoviruses (mHAd.sT and mHAdLyp.sT), the seven hypervariable regions of Ad5 were substituted with the corresponding sequence of Ad48 [6, 16]. For LyP-1 receptor binding adenoviruses (AdLyp.sT and mHAdLyp.sT), a sequence encoding nine amino acids (LyP-1 peptide:CGNKRTRGC) was introduced into the HI loop of viral fiber [6]. All other key components: mutant E1A (01/07), ADP (adenoviral death protein), and ITR (inverted terminal repeats), are sequentially positioned. The construction maps of mHAd.sT, AdLyp.sT and mHAdLyp.sT were published previously [6, 16]. Good Laboratory Practice (GLP) mass productions of viruses for animal studies were prepared by Gene Vector Core of Baylor College of Medicine (Houston, TX). Anti-mouse PD-1 (clone RMP1-14, catalog#: BE0146, lot#: 810421D1) and anti-mouse CTLA-4 (clone 9H10, catalog#: BE0131, lot#: 755621D1) antibodies were purchased from Bio X Cell (Lebanon, NH). All other materials used in this study were purchased from the vendors based on technique requirement and their past performance in the laboratory [6, 12, 16, 25–28].

### Animal studies

All animal experimental procedures were approved by the Institutional Animal Care and Use Committee (IACUC) at the NorthShore University Health System. Because mice would be exposed to adenoviruses, a non-standard animal care routine was adopted. In short, mice were housed in a BSL-2 level biocontainment room with cages labeled accordingly with biohazard signs; soft bedding materials such as shredded filter paper were used throughout the experiment; cages were cleaned routinely and the beddings were autoclaved before disposal. Also, because mice would develop tumors and metastasis, the following measurements were taken for feeding and minimizing animal distress: for cages where some mice couldn’t reach up to access the feed we moistened the regular diet and placed it on the cage floor (in a small dish or on the bedding) or put an in-cage hanging feeder. We also used a water bottle with an extra-long dispenser to make water access a little easier when necessary. In addition, during the course of the experiment, animal health was monitored closely using a body condition (BC) score system combined with other clinical indicators of the distress/clinical severities (body weight, tumor size, degree of ulceration, etc). Any mice reaching criteria for early euthanasia were euthanized immediately and not included in this study. We started with 5 mice per group for each time point of the mechanism study and 6 mice per group for the therapeutic study, and we expected to detect statistically significant differences at 95% confidence level with adequate power while using optimized experimental protocol to increase effect sizes and decrease experimental variations. However, for some groups, because we needed to euthanize mice with severe ulceration of subcutaneous tumor or other health conditions early, or the sample amounts we obtained were not enough to be used for all tests, the sample numbers that were applied to some tests have been reduced to 3 or 4.

### Tumor formation, adenoviral treatments, and sample preparation

To establish mouse mammary tumor syngeneic mouse model, we injected 2 × 10^6^ 4T1 cells per mouse subcutaneously (day 0) into the dorsal right flank of sixty female BALB/c mice (6-8 weeks old), based on our previous experience for this model [6]. The subcutaneous tumors were usually apparent after day 6. On days 7 and 12 post tumor cell inoculation, mHAdLyp.sT, AdLyp.sT, mHAd.sT, Ad.sT, Ad(E-).null, or the vehicle control (PBS buffer) were individually administered intravenously via tail vein (2.5 × 10^10^ VPs or 100 μl of buffer per mouse for each injection, ten mice per treatment group) to the tumor bearing mice. No randomization method was used for allocating animals to different experimental groups, since no significant difference of tumor size on day 7 has ever been observed using our optimized tumor cell inoculation protocol. 2.5 × 10^10^ VPs/mouse/injection is a practical initial dose in the immunocompetent mouse model based on our previous dose escalation studies in the immunodeficient mouse tumor models where adenoviruses were also injected intravenously [6, 16]. Several additional mice that were not injected with any tumor cells and viruses served as the normal control. Mice were monitored carefully every day and some mice with significant stress/sick sign and severely ulcerated tumor were euthanized early according to our animal protocol. On day 14, two days (48 h) after the second viral injection, whole blood (*n* = four to five mice per treatment group) was withdrawn via cardiac puncture in anesthetized animals before they were euthanized to prepare mouse serum and blood cells for toxicity biomarker, circulating cytokine, and flow cytometry analysis, respectively. Then, subcutaneous tumors, liver, lung, and spleen tissues were removed. They were either frozen for qRT-PCR analysis or processed immediately for flow cytometry analysis for selected groups. On day 25, all remaining mice were euthanized to obtain the samples described above and processed accordingly. Liver and spleen tissues were used for all assays but blood and tumor samples were used for a subset of assays depending on the final volumes collected. The sample sizes for each assay are indicated in the relevant figures and figure legends.

### Toxicity studies and blood immune marker analysis

DNA was extracted from liver samples and viral DNA copy numbers were measured by quantitative PCR (qPCR) using the method previously described [6, 15, 16]. In short, liver genomic DNA was extracted by using QIAamp Fast DNA Tissue Kit (catalog#: 51404, Valencia, CA), and Ad5 genome primers (sense: 5′ cagcgtagccccgatgtaag 3′; anti-sense: 5′ tttttgagcagcaccttgca 3′) were used for quantifying viral genomic DNA copy numbers on a StepOnePlus real-time PCR system (Applied Biosystems/Life Technologies, Foster City, CA). Serum lactate dehydrogenase (LDH), alanine transaminase (ALT), and aspartate aminotransferase (AST) levels were measured with serum samples using commercially available kits as described in the previous publications [6, 15, 16]. Blood sTGFβRIIFc and TGFβ-1 expression were measured using the lab ELISA method described in previous publications [6, 12, 15, 16], or a TGFβ-1 ELISA kit from R&D Systems (catalog#: DY1679-05, Minneapolis, MN) following manufacturer’s instruction, respectively. A subset of sera was also submitted for additional cytokine analysis using a MSD custom U-Plex assay kit from PBL Assay Science (Piscataway, NJ) to quantify the serum levels of IFN- γ, TNF-a, IL-2, IL-4, IL-6, IL-10, IL-12p70, IL-17A, and GM-CSF.

### Quantitative real-time RT-PCR analysis of gene expression

Total RNA was isolated from mouse spleen and tumor tissues and cDNA was synthesized using the qScript cDNA SuperMix (catalog#: 101414-102, VWR International, Inc. Radnor, PA) according to the manufacturer’s instructions. mRNA expression profiles of various genes in spleen and tumor tissues were determined by qRT-PCR on the StepOnePlus real-time PCR system (Applied Biosystems/Life Technologies, Foster City, CA). The efficiency and specificity information of all primers used in this study are in Supplementary Table 1. In addition, annealing temperature and concentrations of each primer pair were optimized for each gene to prevent primer-dimer formation. A pair of IDT ReadyMade primers for GAPDH (Forward: catalog#: 51-01-07-12, lot#: 0000723926; Reverse: catalog#: 51-01-07-13, lot#: 0000697763; Integrated DNA Technologies, Coralville, Iowa) was used as the endogenous gene control, and relative expression (RQ-fold changes) of the target genes was calculated using the ΔΔCT method with normal or buffer samples as the calibrators.

### Immune cell analysis by flow cytometry

Single-cell suspensions from blood, spleen, and tumor tissues were prepared by the method described in the previous lab publication [12], and stained to evaluate inflammatory cell types. All antibodies and supplies were purchased from Biolegend (San Diego, CA). T cells were evaluated by: Spark Violet 538 Anti-Mouse CD45 Clone 30-F11 (catalog#: 103180, lot#: B348977 and B329033), APC/Cy7 Anti-Mouse CD4 Clone GK1.5 (catalog#: 100414, lot#: B286275), PE/Cy7 Anti-Mouse CD8a Clone 53-6.7 (catalog#: 100722, lot#: B312604), FITC Anti-Mouse CD25 Clone 3C7 (catalog#: 101908, lot#: B276320), BV711 Rat Anti-Human/Mouse CD44 Clone IM7 (catalog#:103057, lot#: B317259), PE Rat Anti-Mouse CD62L Clone MEL-14 (catalog#: 104408, lot#: B310458), PerCP-Cy5.5 Rat Anti-Mouse CD3 Clone 17A2 (catalog#: 100218, lot#: B314600), and either Alexa Fluor 647 Anti-Mouse IFN-γ Clone XMG1.2 (catalog#: 505814, lot#: B288855) or Alexa Fluor 647 Anti-Mouse Foxp3 Clone MF-14 (catalog#: 126408, lot#: B323458). Manufacturer protocols were followed for cell surface, intracellular, or nuclear transcription factor staining as appropriate. Similarly, myeloid cells were evaluated with Spark Violet 538 Anti-Mouse CD45 Clone 30-F11 (catalog#: 103180, lot#: B348977 and B329033), APC/Cy7 Anti-Mouse Ly-6C Clone HK1.4 (catalog#: 128026, lot#: B309226), PE/Cy7 Anti-Mouse Ly-6G Clone 1A8 (catalog#: 127618, lot#: B322354), FITC Anti-Mouse CD11b Clone M1/70 (catalog#: 101206, lot#: B313038), PE Anti-Mouse CD206 Clone C068C2 (catalog#: 141706, lot#: B290860), PerCP-Cy5.5 Anti-Mouse CD86 Clone GL1 (catalog#: 105028, lot#: B310674), Alexa Fluor 647 Anti-Mouse F4/80 Clone BM8 (catalog#: 123122, lot#: B336655), and BV711 Anti-Mouse CD11c Clone N418 (catalog#: 117349, lot#: B311162). Zombie UV viability dye (catalog#: 423108, lot#: B337299) was used to gate on viable cells. Data were obtained on a BD FACSAria Fusion and analyzed using FlowJo software. Fluorescence minus one controls were used to set gates as needed. Representative images of the gating strategy to capture data for particular cellular populations of interest are shown in the Supplementary Figure.

### Therapeutic analysis

Subcutaneous 4T1 tumors were established in forty-eight female BALB/c mice by the method described above. On day 6 post tumor cell inoculation, tumor dimension was measured (in mm) by using a caliper. Tumor volumes were calculated by the following formula: (width^2^ x length)/2. Then, tumor bearing mice were arbitrarily divided into eight groups, without statistical differences of tumor volume between each group (*n* = 6 per group). On day 6 and 8 post tumor cell inoculation, mHAdLyp.sT or the vehicle control (PBS buffer) was administered intravenously via tail vein (2.5 × 10^10^ VPs or 100 μl of buffer per mouse each time). On days 7, 9, 11, and 13, anti-PD-1 and/or anti-CTLA-4 antibodies were administered intraperitoneally (0.2 mg per mouse per antibody each time) in the groups as indicated in the result section. The tumor volumes were monitored again on days 9, 13, 16, 20, and 23, together with their body weights. Four additional mice that were not injected with any tumor cells served as the normal control for mouse health conditions. Mice were monitored carefully every day for significant stress/sick signs or severely ulcerated tumors according to our animal protocol. On day 25, all remaining mice were euthanized, and the blood, spleen, lung and tumor tissues were collected according to lab procedures [6, 12], and then either processed immediately or frozen for later use. Half of each lung was fixed and prepared for H&E staining according to our published protocol [6, 12]. Lung sections were examined by a Nikon Eclipse TE200 Inverted Microscope. Nikon DS-Fi3 microscope camera and NIS-Elements BR 5.41.02 were used for documenting micrographs. Pulmonary metastatic burden was quantified by using ImageJ software for each lung section with low magnification images, but higher magnification images were used when necessary for identification of micrometastases. Both tumor volume and lung metastatic area were measured blindly by researchers without knowing which animal group.

### Statistical analyses

All statistical analyses were performed using GraphPad Prism software 9 (version 9.3.1 (471)). One-way ANOVA (Kruskal-Wallis or ordinary) with Dunn’s or Bonferroni’s multiple comparisons tests were used for group statistical analyses, and unpaired t tests were used to determine the difference between two categorical groups when required. For outcomes with repeated measurements over time (e.g., tumor volume growth), two-way ANOVA with Tukey’s multiple comparisons tests were used. In addition, F tests were used to compare variances among and between groups and those with similar variance were statistically compared and shown. Significant difference is shown as: *p < 0.05, **p < 0.01, ***p < 0.001, ****p < 0.0001.

## Results

**Fig. 1:**
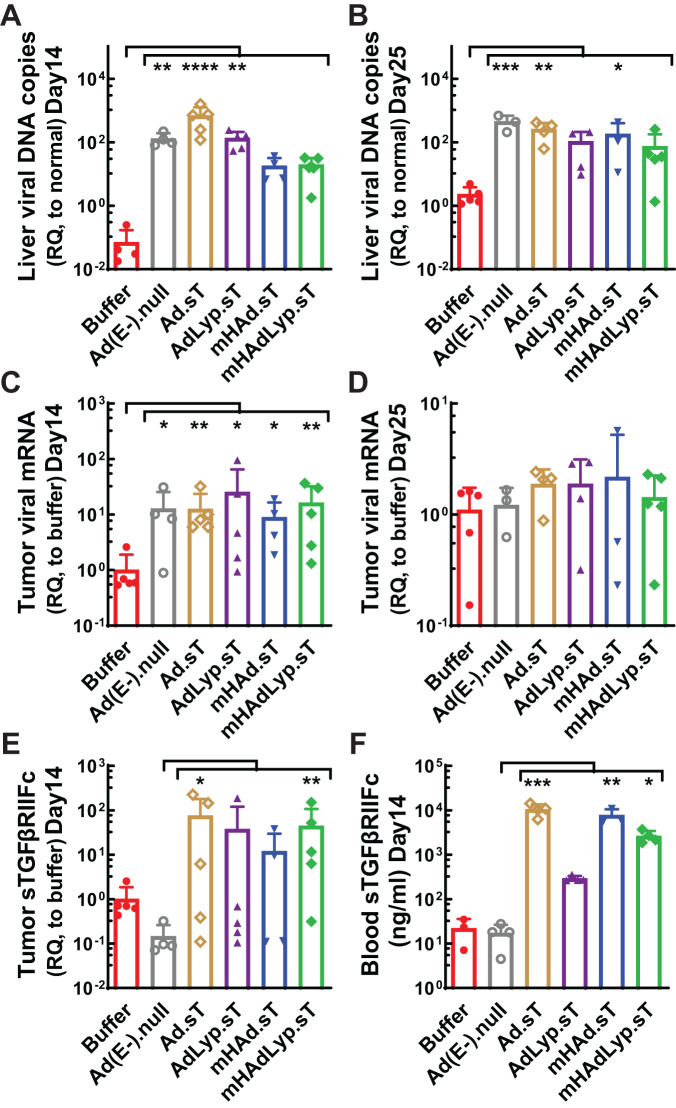
Systemic administration of mHAdLyp.sT in immunocompetent 4T1 mouse model exhibited reduced liver uptake but retained viral gene and sTGFβRIIFc expression in tumor tissues and/or blood serum. **A** Viral DNA copy numbers in the liver on day 14 of tumor cell inoculation were measured and shown (*n* = 4 or 5 for each group). **B** Viral DNA copy numbers in the liver on day 25 were measured and shown (*n* = 3 to 5 for each group). **C** Viral genome expression in the tumor on day 14 was measured by qRT-PCR and shown with RQ-fold changes to the buffer group (*n* = 4 or 5 for each group). **D** Viral genome expression in the tumor on day 25 were measured and shown (*n* = 3 to 5 for each group). **E** sTGFβRIIFc expression in the tumor on day 14 was measured by qRT-PCR and shown (*n* = 4 or 5 for each group). **F** sTGFβRIIFc levels in mouse serum on day 14 were measured by ELISA and shown (*n* = 3 or 4 for each group). Significant differences (compared to the buffer and/or Ad(E-).null by One-Way ANOVA with Dunn’s multiple comparisons tests) are shown as: *p < 0.05, **p < 0.01, ***p < 0.001, or ****p < 0.0001.

We previously reported that systemic administration of mHAdLyp.sT in immunodeficient nude mice resulted in reduced uptake in the liver and spleen, reduced hepatotoxicity and systemic toxicity, and attenuated innate immune response [6]. In this study we used immunocompetent BALB/c mice bearing 4T1 TNBC tumors to determine viral tropism, toxicity, and immune responses by the systemic administration of mHAdLyp.sT. First of all, liver samples collected 48 h after adenovirus injection were evaluated for viral genomic DNA copy numbers. Unlike the control vectors lacking hexon modification for liver-de-targeted tropism (AdLyp.sT, Ad.sT and Ad(E-).null), mHAdLyp.sT treatment didn’t lead to a significant increase of liver viral genomic DNA copy numbers compared to the buffer group (Fig. 1A). mHAdLyp.sT treatment also did not significantly increase viral uptake in liver on day 25, although a significant increase of liver viral DNA remained in the Ad.sT and Ad(E-).null groups (Fig. 1B). Thus, we concluded that mHAdLyp.sT had reduced liver uptake in immunocompetent mice as well. We further evaluated the tumor viral uptake by qRT-PCR of viral genome and sTGFβRIIFc expression. We observed significant viral genome tumor expression for all of our adenoviruses (AdLyp.sT, mHAd.sT and Ad(E-).null vs buffer: *P* < 0.05; mHAdLyp.sT and Ad.sT vs buffer: *P* < 0.01) in day 14 tumor samples (Fig. 1C), although these changes were no longer observed in day 25 tumor samples (Fig. 1D). We also found that mHAdLyp.sT and Ad.sT treatment led to a significant increase in sTGFβRIIFc mRNA expression when compared to the Ad(E-).null treated group (mHAdLyp.sT: *P* < 0.01; Ad.sT: *P* < 0.05) in day 14 tumor samples (Fig. 1E). In addition, a significant increase of blood sTGFβRIIFc protein expression was observed in serum samples on day 14 for mHAdLyp.sT, mHAd.sT, and Ad.sT (Fig. 1F, *P* < 0.05, 0.01, or 0.001 vs Ad(E-).null, respectively). These data suggest that mHAdLyp.sT maintained tumor tropism in 4T1 mouse TNBC tumors but had reduced liver uptakes when administered systemically.

**Fig. 2:**
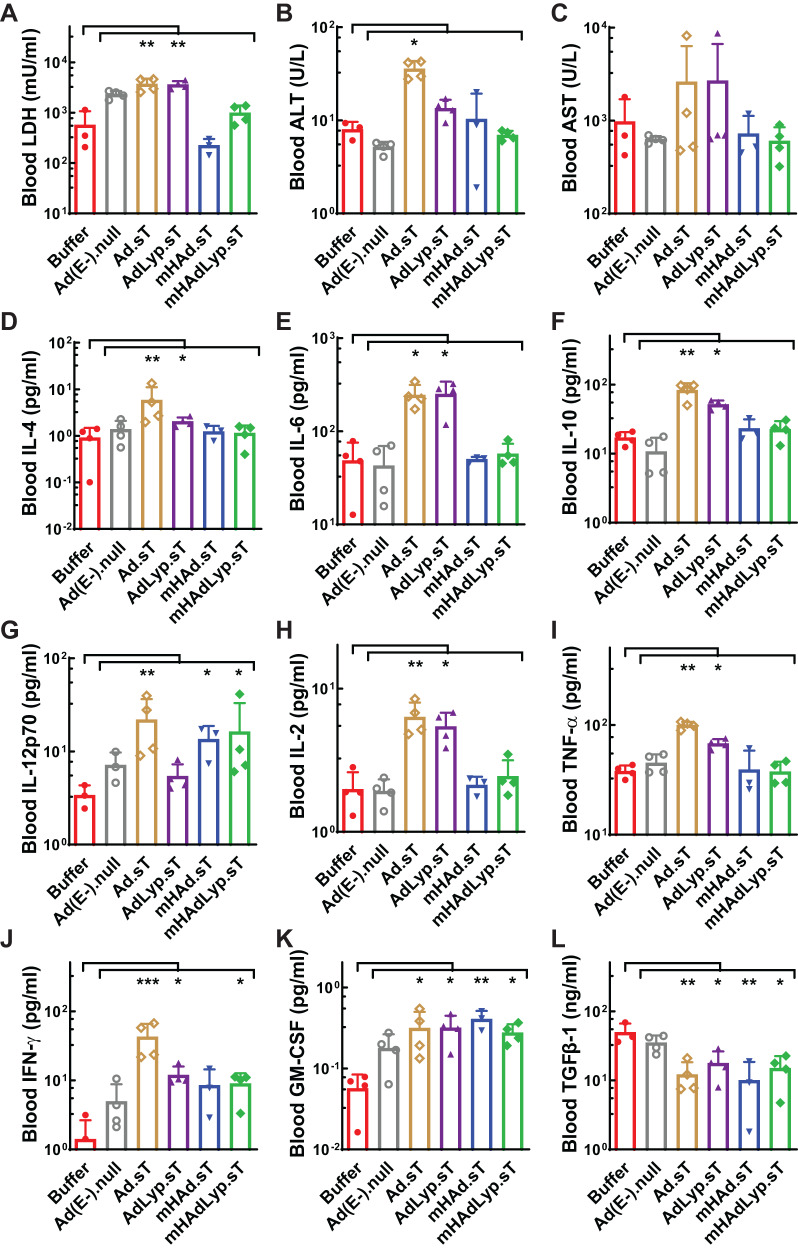
mHAdLyp.sT produced reduced hepatic and systemic toxicity, and reduced proinflammatory cytokine responses. **A** Blood LDH levels on day 14 were measured to indicate systemic toxicity. **B, C** Blood ALT and AST tests on day 14 were showed for hepatic toxicity analysis. **D**–**L** Analysis of cytokines (IL-4, IL-6, IL-10, IL-12p70, IL-2, TNF-a, IFN- γ, GM-CSF, and TGFβ-1) in day 14 serum samples was shown. *n* = 3 or 4 for each group. Significant differences (compared to the buffer and/or Ad(E-).null by One-Way ANOVA with Dunn’s multiple comparisons tests) are shown as: *p < 0.05, **p < 0.01, or ***p < 0.001.

We used mouse sera obtained 48 h after adenovirus injection to examine the short-term hepatotoxicity, systemic toxicity, and systemic inflammatory responses in this 4T1 syngeneic mouse model. The quantification of serum LDH, ALT, and AST levels suggested that the systemic administration of mHAdLyp.sT and another adenovirus containing Ad5/48 chimeric hexon (mHAd.sT) resulted in no significant systemic and hepatic toxicity (Fig. 2A–C). Also, while the replicating adenovirus with Ad5 hexon (Ad.sT) stimulated a significant increase of both Th2 cytokines (IL-4, IL-6, and IL-10) (Fig. 2D–F, Ad.sT vs buffer: *P* < 0.05 or 0.01) and Th1 cytokines (IL-12p70, IL-2, TNF-α and IFN-γ) (Fig. 2G, J, Ad.sT vs buffer: *P* < 0.01 or 0.001), mHAdLyp.sT only elicited the significant increase of IL-12p70 and IFN-γ Th1 cytokines (Fig. 2G, J, mHAdLyp.sT vs buffer: *P* < 0.05). For the other two adenoviruses we tested (AdLyp.sT and mHAd.sT), AdLyp.sT is similar to Ad.sT but mHAd.sT seems less effective in eliciting Th1 cytokine response than mHAdLyp.sT as only a significant change in IL-12p70 was detected (Fig. 2G, mHAd.sT vs buffer: *P* < 0.05). To strengthen these findings, comparison of the toxicity profiles between mHAdLyp.sT and other tested adenoviruses or the buffer group is summarized in Supplementary Table 2. mHAdLyp.sT has significantly lower levels of LDH, ALT, IL-4, IL-6, IL-10, IL-2, and TNF-α than Ad.sT while having higher levels of IL-12p70 and IFN-γ than the untreated group. Severe systemic toxicity and inflammatory responses to adenoviruses upon systemic delivery is a major obstacle for their potential clinical application [13]. Thus, mHAdLyp.sT is more likely to be applicable for future clinical trials as it didn’t induce detectable hepatotoxicity and systemic toxicity but maintained some critical anti-tumor Th1 cytokine response such as IL-12p70 and IFN-γ.

We also tested serum GM-CSF and TGFβ-1 levels on day 14 to examine changes in these immunosurveillance molecules where different expression levels can either promote anti-tumor immune responses (GM-CSF) or lead to immunosuppression (TGFβ-1) [10, 29, 30]. All replicating adenoviruses expressing sTGFβRIIFc almost equally prompted GM-CSF production while hindering immune inhibitory TGFβ-1 secretion (Fig. 2K, L). It is not surprising to see reduced TGFβ-1 levels in mouse sera by these therapeutic adenoviruses since they all express sTGFβRIIFc that can bind TGFβ-1 and neutralize its downstream signaling events via Type II receptors, but the increase of serum GM-CSF levels are also encouraging because many GM-CSF based treatment strategies are currently used in the clinic or hold promise in clinical trials for several cancer types, including breast cancer. Additionally, the expression levels of these biomarkers in sera obtained on day 25 were also analyzed, but the results did not support persistent changes (data not shown).

**Fig. 3:**
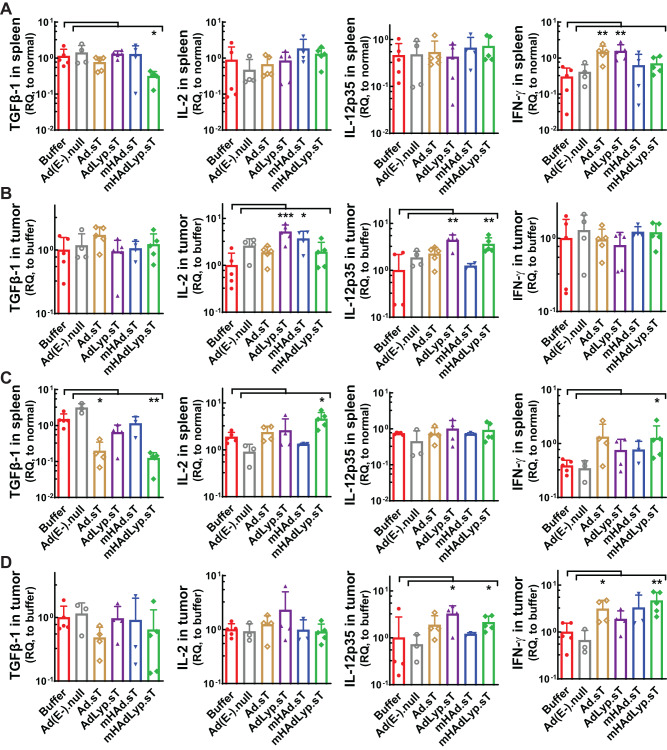
mHAdLyp.sT favored the production of Th1 cytokines (IL-2, IL-12, IFN-γ) rather than Th2 cytokines (TGFβ-1) systemically and in the tumor. **A** TGFβ-1, IL-2, IL-12, and IFN-γ expression in spleen on day 14 was analyzed by qRT-PCR and shown. **B** Day 14 tumor expression of TGFβ-1, IL-2, IL-12, and IFN-γ expression was measured and shown. **C** Day 25 spleen expression of TGFβ-1, IL-2, IL-12, and IFN-γ expression was shown. **D** Shown was day 25 tumor expression of TGFβ-1, IL-2, IL-12, and IFN-γ expression. Panels labeled A and C are the same set of analysis for spleen samples obtained from a different ending point (Day 14 or Day 25), and panels labeled (**B**, **D**) are data of the same set of targets in tumor samples from either Day 14 or Day 25. For day 14 samples in (**A**, **B**), *n* = 4 or 5 for each group; for day 25 samples in (**C**, **D**), *n* = 3–5. Significant differences (compared to the buffer group by One-Way ANOVA with Dunn’s or Bonferroni’s multiple comparisons tests) are shown as: *p < 0.05, **p < 0.01, or ***p < 0.001.

In tumor-bearing mice, spleen is the major immunomodulatory organ and tumor is where all actions are propelled. Therefore, we analyzed the expression levels of the biomarkers above on both day 14 and day 25 samples of spleen and tumor by qRT-PCR. Only mHAdLyp.sT inhibited TGFβ-1 expression in spleen on both day 14 and day 25 (Fig. 3A, day 14; Fig. 3C, day 25, both the first panel, *P* < 0.05 or 0.01 vs buffer). We also analyzed the expression of other Th2 cytokines besides TGFβ-1 (IL-4 and IL-6) in spleen and tumor samples, but treatment with mHAdLyp.sT didn’t lead to any significant changes when compared to the buffer group (data not shown). For Th1 cytokines of interest (IL-12, IL-2, TNF-α and IFN-γ), in spleen, we detected increased levels of IL-2 and IFN-γ expression by mHAdLyp.sT treatment on day 25 (Fig. 3C, day 25; IL-2, the second panels; IFN-γ, the fourth panels; both *P* < 0.01 vs buffer). It is interesting that no significant changes of them were observed in the mHAdLyp.sT treatment group in day 14 spleen samples (Fig. 3A, the second to fourth panels), suggesting mHAdLyp.sT may be more likely to have delayed but persistent systemic immunomodulatory effects in spleen. In the tumor microenvironment, mHAdLyp.sT treated group had increased expression of IL-12 on both day 14 and day 25, and IFN-γ on day 25 (Fig. 3B, day 14; Fig. 3D, day 25; IL-12, the third panels; IFN-γ, the fourth panels; *P* < 0.05 or 0.01 vs buffer). No significant changes of TNF-α in both spleen and tumor was seen at any of our ending points (data not shown), but the localized stimulation of two critical Th1 cytokines (IL-12 and IFN-γ) in tumors by mHAdLyp.sT suggests that mHAdLyp.sT is able to help prime antitumor immunity directly in the tumor, specifically with IL-12, which was elevated by mHAdLyp.sT at both early and late stages of tumor resistance.

**Fig. 4:**
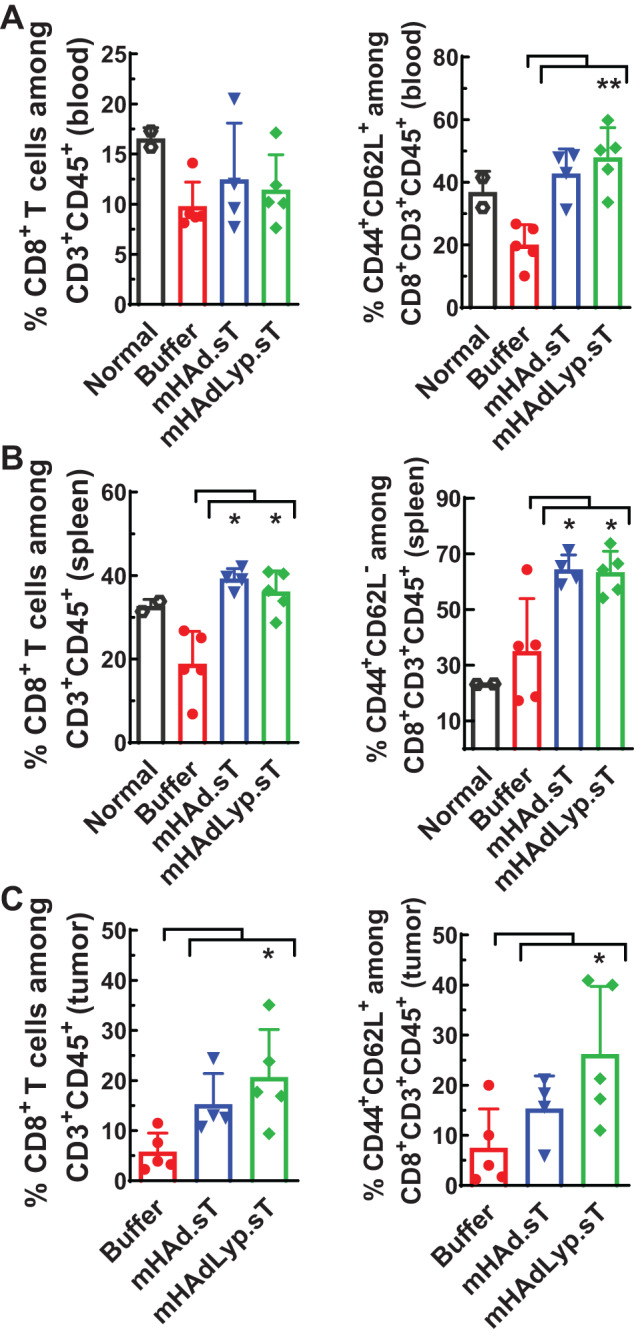
mHAdLyp.sT increased CD8^+^ T lymphocytes and/or CD8^+^ memory cells systemically and in the tumor on day 14. **A** The percentages of CD8^+^ T cells and CD8^+^ central memory cells among T cells in blood were analyzed by flow cytometry and shown. **B** The percentages of CD8^+^ T cells and CD8^+^ effector memory cells in spleen were shown. **C** Shown were the percentages of CD8^+^ T cells and CD8^+^ central memory cells in tumor samples. Panel (**A**–**C**) represents the results from blood, spleen, or tumor tissue, respectively. *n* = 4 or 5 for the buffer and the treatment group; *n* = 2 for the normal group. Significant differences (compared to the buffer group by One-Way ANOVA with Dunn’s or Bonferroni’s multiple comparisons tests) are shown as: *p < 0.05, or **p < 0.01.

Next, we profiled the immune cell population changes in blood, spleen, and tumor samples from both day 14 and day 25 samples. On day 14 of blood samples, mHAdLyp.sT treatment led to a significant increase in the percentage of central memory cells (T_CM_, CD44^+^CD62L^+^) among CD8^+^ T lymphocytes (Fig. 4A, the right panel, *P* < 0.01 vs buffer), even though the CD8^+^ proportion of total T cells remained similar in blood (Fig. 4A, the left panel). In spleen and tumor, we observed significantly elevated percentages of CD8^+^ T lymphocytes by mHAdLyp.sT (Fig. 4B, C, the left panels, *P* < 0.05 vs buffer). The control adenovirus we used in the flow studies, mHAd.sT, only increased the percentage of CD8^+^ T cells in spleen (Fig. 4B, the left panel). Importantly, in tumor, mHAdLyp.sT treatment also increased the percentage of CD8^+^ central memory cells (T_CM_, CD44^+^CD62L^+^) significantly on day 14 (Fig. 4C, the right panel, *P* < 0.05 vs buffer). Interestingly, in spleen, the percentage of CD8^+^ effector memory cells (T_EM_, CD44^+^CD62L^−^) was raised significantly by both adenoviruses (Fig. 4B, the right panel, *P* < 0.05 vs buffer). In mouse models, both CD8^+^ T_CM_ and T_EM_ cells are vital components of the anti-tumor response [31]. Based on these data mHAdLyp.sT may depend on different microenvironments of specific tissues to help CD8^+^ T cells acquire distinct anti-tumor memory cell characteristics.

**Fig. 5:**
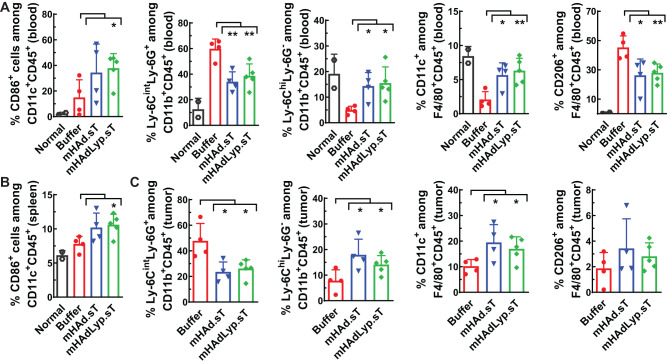
mHAdLyp.sT treatment led to myeloid cell remodeling that favors their anti-tumor functions both systemically and in the tumor on day 14. **A** The percentages of CD86^+^ dendritic cells (DCs), g-MDSCs (Ly-6C^int^Ly6G^+^), m-MDSCs (Ly-6C^hi^Ly6G^−^), M1 macrophages (CD11c^+^F4/80^+^), and M2 macrophages (CD206^+^F4/80^+^) in blood were analyzed by flow cytometry and shown. **B** The percentage of CD86^+^ DCs in spleen on day 14 was shown. **C** Shown were the percentages of g-MDSCs (Ly-6C^int^Ly6G^+^), m-MDSCs (Ly-6C^hi^Ly6G^−^), M1 macrophages (CD11c^+^F4/80^+^), and M2 macrophages (CD206^+^F4/80^+^) in tumor samples. Panel (**A**–**C**) represents the results from blood, spleen, or tumor tissue, respectively. *n* = 4 or 5 for the buffer and the treatment group; *n* = 2 for the normal group. Significant differences (compared to the buffer group by unpaired t-tests) are shown as: *p < 0.05, or **p < 0.01.

We also conducted flow analysis for various myeloid cell populations on day 14 samples. CD86 is a key target for CTLA-4 immune regulation and is important for T cell activation and survival [32]. In blood, the mHAdLyp.sT treated group had a significant increase in CD86^+^ dendritic cells (DCs) (CD86^+^CD11C^+^) on day 14 (Fig. 5A, the left panel, *P* < 0.05 vs buffer). The increased percentage of DCs was also observed in spleen by mHAdLyp.sT treatment (Fig. 5B, *P* < 0.05 vs buffer). For myeloid-derived suppressor cells (MDSCs), both adenoviruses led to a decrease of in granulocytic/polymorphonuclear MDSCs (g-MDSCs, Ly6C^int^Ly6G^+^CD11b^+^, Fig. 5A, the second panel, *P* < 0.01 vs buffer) and an increase in monocytic MDSCs (m-MDSCs, Ly6C^hi^Ly6G^-^CD11b^+^, Fig. 5A, the third panel, *P* < 0.05 vs buffer) in blood. Similar changes in MDSCs subpopulations were also detected in tumor samples (Fig. 5C, the first two panels, *P* < 0.05 vs buffer). Both g-MDSCs and m-MDSCs are immune suppressive. However, mHAdLyp.sT treatment favors predominantly m-MDSCs, which employs nitric oxide (NO) and immunosuppressive cytokines/molecules such as IL-10, TGFβ-1, and PD-L1 to mediate immune suppression [33]. Since mHAdLyp.sT inhibits the TGFβ signaling pathway, a combination therapy with immune checkpoint inhibitors could more effectively inhibit MDSCs related immune suppression, both systemically and in the tumor.

Polarizing macrophages towards a pro-inflammatory and tumor-inhibiting M1 phenotype is considered another important sign of immune-inflamed response by potential immunotherapy agents [34]. Both mHAd.sT and mHAdLyp.sT treatment significantly increased M1 macrophage (CD11C^+^) percentages among F4/80^+^CD45^+^ cells in blood and tumor samples (Fig. 5A, blood, the fourth panel; Fig. 5C, tumor, the third panel, *P* < 0.01 or *P* < 0.05 vs buffer). Both adenoviruses also significantly reduced the percentage of M2 macrophages (CD206^+^) in blood (Fig. 5A, the last panel, *P* < 0.01 or *P* < 0.05 vs buffer). Noteworthy, mHAdLyp.sT seems to be more effective in macrophage polarization towards a cancer cell-killing phenotype systemically than mHAd.sT since changes in both M1 and M2 macrophages in blood by mHAdLyp.sT were more significant than those by mHAd.sT, when they were compared to the buffer group (Fig. 5A, the last two panels; mHAdLyp.sT vs buffer, *P* < 0.01; mHAd.sT vs buffer, *P* < 0.05).

**Fig. 6:**
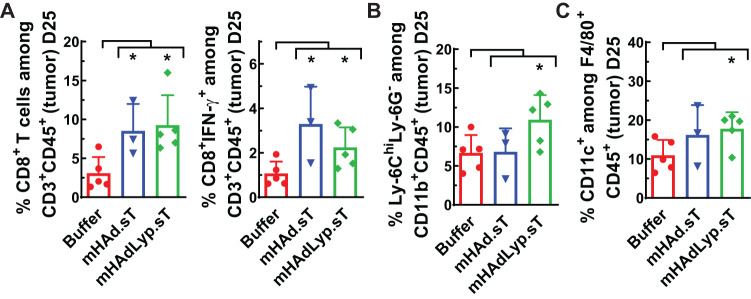
On day 25, anti-tumor immune cell changes by adenoviruses were sustained in the tumor. **A** The percentages of CD8^+^ T cells and CD8^+^ IFN- γ^+^ T cells in tumor on day 25 were analyzed by flow cytometry and shown. **B** The percentage of m-MDSCs (Ly-6C^hi^Ly6G^−^) among CD11b^+^ cells in tumor on day 25 was shown. **C** Shown was the percentage of M1 macrophages among F4/80^+^ cells in tumor on day 25. *n* = 3 or 5 for each group. Panel (**A**–**C**) represents the data from analysis of T cells, MDSCs, and macrophages in the tumor tissue, respectively. Significant differences (compared to the buffer group by unpaired t tests) are shown as: *p < 0.05.

The same set of immune cell analyses were conducted with the Day 25 samples of blood, spleen and tumor. We did not detect any meaningful differences systemically at this late tumor resistant stage (data not shown). However, an immune inflamed tumor microenvironment seems largely retained in adenovirus treated groups, especially for those with mHAdLyp.sT treatment. Within tumors, the percentage of CD8^+^ T lymphocytes and IFN-γ producing CD8^+^ T cells were both significantly increased by adenovirus treatments (Fig. 6A, *P* < 0.05 vs buffer). Furthermore, only mHAdLyp.sT led to a significant increase of m-MDSCs (Ly6C^hi^Ly6G^-^CD11b^+^, Fig. 6B, *P* < 0.05 vs buffer) and M1 macrophage (CD11C^+^F4/80^+^, Fig. 6C, *P* < 0.05 vs buffer) in day 25 tumors. Taken together, our immune cell analysis data in the tumor-bearing immunocompetent mouse model supports mHAdLyp.sT as a potent primer towards the anti-tumor phenotype that could synergize with other immunomodulators to achieve better therapeutic results.

**Fig. 7:**
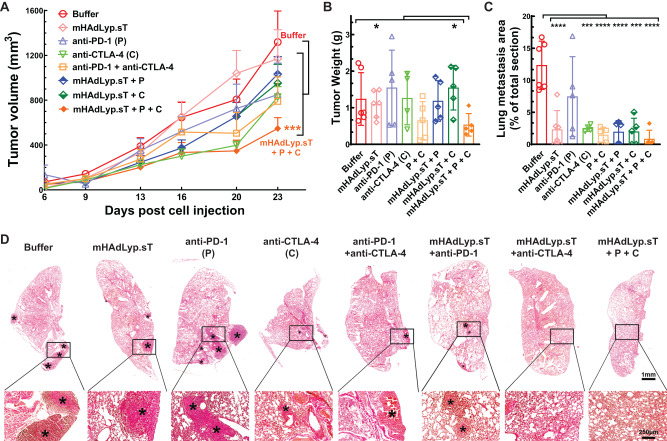
A pilot therapeutic evaluation of mHAdLyp.sT and its combination with ICIs in the 4T1 model showed the triple combination was most effective in inhibiting tumor growth and lung metastasis. **A** The primary 4T1 tumor growth was measured by tumor volume for 23 days post cell injection. **B** Weights of primary tumors at the terminal point were shown (Day 25). **C** Shown was microscopic lung metastasis analysis. **D** Representative images of H&E-stained lung sections of the buffer and each treatment group were shown. *Indicates the site of lung micrometastases and macrometastases. Magnification is indicated by scale bars (the scale bar for low magnification images is 1 mm; that for high magnification is 250 µm). *n* = 4 to 6 for each group. Significant differences by two-way ANOVA (**A**), t-tests (**B**), or one-way ANOVA with Bonferroni’s multiple comparisons tests (**C**) are shown as: *p < 0.05, **p < 0.01, ***p < 0.001, or ****p < 0.0001.

In the past, we showed that systemic administration of mHAdLyp.sT inhibited bone metastases in a human TNBC cell line (MDA-MB-231) immunodeficient mouse model. Therefore, we applied mHAdLyp.sT alone and together with immune checkpoint inhibitors (ICIs) to test their treatment efficacy in this 4T1 immunocompetent model. Our tumor volume analysis indicated that the combination of mHAdLyp.sT, anti-PD-1 and anti-CTLA-4 antibodies (triple treatment) was the only group that significantly inhibited primary tumor progression when compared to the buffer group using a two-way ANOVA analysis at the end of this experiment (Day 23) (Fig. 7A, *P* < 0.001 vs buffer). This was further supported by tumor weight analysis because the average tumor weight of the triple treatment group was lowest and was significantly different from two other groups by t tests (Fig. 7B, *P* < 0.05 vs mHAdLyp.sT and mHAdLyp.sT+anti-CTLA-4). Most importantly, all treatment groups, except for anti-PD-1 alone, were almost equally effective in inhibiting lung metastasis by our H&E staining microscopy analysis (Fig. 7C, *P* < 0.001 or *P* < 0.0001 vs buffer; Fig. 7D, representative images of H&E-stained lung sections). Although mHAdLyp.sT didn’t alleviate the growth of the primary tumor, no significant difference in inhibiting lung metastasis between mHAdLyp.sT alone and the triple treatment when comparing them directly by t test was observed. Because mHAdLyp.sT is much safer to be used systemically, currently we are working on new treatment experiments with increased doses of mHAdLyp.sT to enhance its anti-tumor activities in this model.

## Discussion

We report here that the new liver-de-targeted TGFβ signaling inhibiting adenovirus: mHAdLyp.sT is safe to be used in the TNBC tumor-bearing immunocompetent mouse model and retains its tumor tropism while applied systemically. mHAdLyp.sT is also potent in stimulating Th1 cytokine production and priming an immune inflamed phenotype, as supported by our cytokine, qRT-PCR and flow cytometry analysis. Thus, mHAdLyp.sT could restore anti-tumor immune responses in highly immunosuppressive cancers, such as mTNBC, and be used synergistically with other systemic immunotherapy approaches, such as ICIs and CAR T cells, etc.

There are several distinct advantages for using mHAdLyp.sT as a potential new systemic treatment option for metastatic cancers. First of all, compared to several FDA-approved oncolytic virus approaches and most of others in the current development [35, 36], mHAdLyp.sT can be safely delivered systemically because it has reduced hepatotoxicity and systemic toxicity. We previously reported that mHAdLyp.sT was safer to use than our other adenoviruses in an immunodeficient mouse model [6], and now in this study we confirm with quantitative analysis that it also results in reduced liver uptake and reduced hepatic and systemic toxicity and proinflammatory cytokine responses in the TNBC tumor-bearing immunocompetent mouse model. The results from the current study are more valuable for preclinical evaluation than those we observed in the previous immunodeficient mouse model since adaptive anti-tumor immune responses are also present in the tumor-bearing immunocompetent mouse model. In addition, because mHAdLyp.sT expresses Ad48 Hexon at HVRs (1-7), which is the most dominant anti-Ad5 antibody epitopes [13], mHAdLyp.sT is expected to circumvent pre-existing neutralizing Ad5 antibodies in human sera. We will carefully screen human serum samples to test if mHAdLyp.sT will be neutralized in the presence of anti-adenoviral antibodies in our future pre-clinical studies.

Secondly, mHAdLyp.sT is engineered with two levels of tumor selective replication/targeting abilities. On one side, the viral backbone of our Ad series adenoviruses only allows them to selectively replicate in human cancer cells but can induce cell lysis for all tumors from different species when used with a high dose [21–26]. On the other side, mHAdLyp.sT has higher binding affinity to tumor and tumor tissues expressing LyP-1 receptors via the LyP-1 peptide sequence inserted into the adenoviral fiber [6]. LyP-1 receptor expression has been shown to be present in many cancers, including breast, prostate, melanoma, glioblastomas, and pancreas, etc. Therefore, mHAdLyp.sT -induced cell death would release tumor-related antigens, such as tumor-associated antigens (TAAs), pathogen- or damage-associated molecular patterns (PAMPs and DAMPs), etc, and trigger antitumor immunity specifically [6, 37].

Third, systemic delivery of mHAdLyp.sT will produce a large amount of sTGFβRIIFc, the TGFβ decoy, both systemically and in the tumor. sTGFβRIIFc will bind with TGFβ to inhibit aberrant TGFβ signaling, relieving TGFβ induced immune suppression. Besides TNBCs, aberrant TGFβ signaling has also been shown to promote tumor growth and metastases of many other cancers, including prostate, kidney, and gastrointestinal cancers [4, 38]. More importantly, aberrant TGFβ signaling has been identified as the key mediator for immune evasion in late stage cancers and their poor responses to cancer immunotherapy [10]. Thus, we expect that the combination therapy with mHAdLyp.sT and ICIs would be able to augment the response rate in immunogenic tumors, and make immune-suppressed tumors responsive to immunotherapy, even in patients with a highly immunosuppressive phenotype. To be noted, we also found that mHAdLyp.sT alone can increase serum levels of GM-CSF and some critical anti-tumor Th1 cytokines such as IL-12 and IFN-γ, and boost the percentage of cytotoxic CD8 + T cells in the spleen and tumor. Especially for IL-12 and IFN-γ, both of which are major determinants of CAR T-cell-based antitumor activity [39, 40]. Thus, these results suggest that mHAdLyp.sT may be able to increase the efficacy of adoptively transferred TCR-transduced or CAR-transduced T cells against metastatic cancers as well, by inducing tumor eradication rather than simply slowing the growth. Our preliminary therapeutic data in 4T1 TNBC mouse model in this study showed encouraging results, although we did not observe complete remission of the 4T1 tumor, which is notoriously non-immunogenic and difficult to treat. Since viral toxicity is the major barrier in the systemic delivery of oncolytic adenoviruses, for mHAdLyp.sT, we will be able to apply it with a higher dose due to indications of low toxicity in this study, and hopefully we will observe enhanced therapeutic efficacy in the future. Particularly, since we didn’t observe the sustained expression of the viral genome and sTGFβRIIFc in the late-stage tumors with the current dose, although the changes of some anti-tumor cytokines and immune cell populations remained, as shown in Fig. 3 panel C and D, and Fig. 6, it would still be very beneficial to try higher doses or repeated doses of mHAdLyp.sT injection in our future studies. In addition, since both aberrant TGFβ signaling and LyP-1 receptor expression are presented in several cancer types, mHAdLyp.sT-based immunotherapy approaches have the potential of targeting several other malignancies, too.

To be noted, TNBCs are a group of highly heterogeneous and fundamentally different diseases with distinct histologic, genomic, and immunologic profiles, which are concentrated under the operational term that stemmed from the fact that they don’t have estrogen receptor (ER), progesterone receptor (PR), and human epidermal growth factor receptor 2 (HER-2) expression [41]. But for late stages of TNBCs, such as mTNBC. a shift towards more immunosuppressive setting has often been observed [1, 3, 36], As a result, the response to ICIs for mTNBCs generally are low (5% if selection for PD-L1 positivity was not used) [3, 36]. The major focus of many current pre-clinical combination immunotherapy trials is to induce a more immune-inflamed phenotype for better outcomes. In this study, a mouse TNBC (4T1) model was used. The 4T1 tumor, although difficult to be treated, is ideal for pre-clinical evaluation of mTNBCs immunotherapies, because it resembles several key genome, transcriptome, and immunome signatures of human mTNBCs [42]. We will further explore signature changes of mHAdLyp.sT and its combination with ICIs by using large-scale data, such as RNA-Seq of the whole transcriptome and single-cell mass cytometry (CyTOF) of immune cell subsets in blood, spleen, and tumor tissues of this model in the future. These studies will not only further confirm the discoveries we reported here, but also give us more information about other important alterations of functional significance, including extracellular matrix genes, tumor vasculature, metabolic pathways, epithelial-mesenchymal transition (EMT), and other pro-metastases genes, besides inflammatory and immune signature profiles systemically and in the tumor. In the current study, we have observed a shift from a Th2 to Th1 effector phenotype, an increase in the frequency of CD8^+^ T cells, an increase in CD8^+^ effector memory cells, an increase in CD86^+^ DCs, an increase in m-MDSCs with a decrease of g-MDSCs, and an increase in cancer cell-killing M1 macrophages. We have not investigated some other important cell types such as Tregs, TCRγδ cells, Th17 cells, natural killer (NK) T cells, and tumor-infiltrating B cells. We realize the enormously complex nature of the inflammatory milieu, and several important studies such as TCR sequencing to determine alterations of T-cell repertoire within the tumor microenvironment should be performed in the future as well [43–45]. In the end, all of our findings in the 4T1 models should be verified in our future studies with TNBC patient samples. To do so, first we will screen TNBC patient samples for all important immune signatures to see how it can guide us for possible future clinical trials with mHAdLyp.sT and its combinations. As we move into the commercial development of mHAdLyp.sT in the future, we should be ready to select patients with proper phenotypic characteristics and molecular features to optimize treatment strategies for mTNBC.

In conclusion, our studies described here are critical to bring forward our novel LyP-1 modified Ad5/48 chimeric hexon oncolytic virus mHAdLyp.sT targeting TGFβ, in combination with immune checkpoint inhibitors, for clinical evaluation in TNBC patients in the future. We are confident that mHAdLyp.sT based combination therapy has the real potential to produce effective immune responses in mTNBC patients who are generally non-responders or respond poorly to current treatments.

### Supplementary information

Supplemental material merged

Supplementary tables

## Data Availability

All data generated and/or analyzed during this study are included and/or mentioned in the manuscript. The datasets are available from the corresponding author on request.

## References

[1] Valencia GA, Rioja P, Morante Z, Ruiz R, Fuentes H, Castaneda CA (2022). Immunotherapy in triple-negative breast cancer: A literature review and new advances. World J Clin Oncol.

[2] Tarantino P, Corti C, Schmid P, Cortes J, Mittendorf E, Rugo H (2022). Immunotherapy for early triple negative breast cancer: research agenda for the next decade. npj Breast Cancer.

[3] Li L, Zhang F, Liu Z, Fan Z (2023). Immunotherapy for Triple-Negative Breast Cancer: Combination Strategies to Improve Outcome. Cancers (Basel).

[4] Liu S, Ren J, Dijke P (2021). Targeting TGFβ signal transduction for cancer therapy. Sig Transduct Target Ther.

[5] Yi M, Li T, Niu M, Wu Y, Zhao Z, Wu K (2022). TGF-β: A novel predictor and target for anti-PD-1/PD-L1 therapy. Front Immunol.

[6] Xu W, Yang Y, Hu Z, Head M, Mangold KA, Sullivan M (2020). LyP-1-Modified Oncolytic Adenoviruses Targeting Transforming Growth Factor β Inhibit Tumor Growth and Metastases and Augment Immune Checkpoint Inhibitor Therapy in Breast Cancer Mouse Models. Hum Gene Ther.

[7] Zhang M, Wu J, Mao K, Deng H, Yang Y, Zhou E (2017). Role of transforming growth factor-β1 in triple negative breast cancer patients. Int J Surg.

[8] Xu X, Zhang L, He X, Zhang P, Sun C, Xu X (2018). TGF-β plays a vital role in triple-negative breast cancer (TNBC) drug-resistance through regulating stemness, EMT and apoptosis. Biochem Biophys Res Commun.

[9] Majidpoor J, Mortezaee K (2021). The efficacy of PD-1/PD-L1 blockade in cold cancers and future perspectives. Clin Immunol.

[10] Batlle E, Massagué J (2019). Transforming growth Factor-β signaling in immunity and cancer. Immunity.

[11] Li Y, Zhang H, Merkher Y, Chen L, Liu N, Leonov S (2022). Recent advances in therapeutic strategies for triple-negative breast cancer. J Hematol Oncol.

[12] Yang Y, Xu W, Peng D, Wang H, Zhang X, Wang H (2019). An Oncolytic Adenovirus Targeting Transforming Growth Factor β Inhibits Protumorigenic Signals and Produces Immune Activation: A Novel Approach to Enhance Anti-PD-1 and Anti-CTLA-4 Therapy. Hum Gene Ther.

[13] Li L, Liu S, Han D, Tang B, Ma J (2020). Delivery and biosafety of oncolytic virotherapy. Front Oncol.

[14] Ban W, Guan J, Huang H, He Z, Sun M, Liu F (2022). Emerging systemic delivery strategies of oncolytic viruses: A key step toward cancer immunotherapy. Nano Res.

[15] Zhang Z, Krimmel J, Zhang Z, Hu Z, Seth P (2011). Systemic delivery of a novel liver-detargeted oncolytic adenovirus causes reduced liver toxicity but maintains the antitumor response in a breast cancer bone metastasis model. Hum Gene Ther.

[16] Xu W, Zhang Z, Yang Y, Hu Z, Wang CH, Morgan M (2014). Ad5/48 hexon oncolytic virus expressing sTGFβRIIFc produces reduced hepatic and systemic toxicities and inhibits prostate cancer bone metastases. Mol Ther.

[17] Roberts DM, Nanda A, Havenga MJ, Abbink P, Lynch DM, Ewald BA (2006). Hexon-chimaeric adenovirus serotype 5 vectors circumvent pre-existing anti-vector immunity. Nature.

[18] Fausther-Bovendo H, Kobinger GP (2014). Pre-existing immunity against Ad vectors: humoral, cellular, and innate response, what’s important?. Hum Vaccin Immunother.

[19] Fogal V, Zhang L, Krajewski S, Ruoslahti E (2008). Mitochondrial/cell-surface protein p32/gC1qR as a molecular target in tumor cells and tumor stroma. Cancer Res.

[20] Song N, Zhao L, Zhu M, Zhao J (2019). Recent progress in LyP-1-based strategies for targeted imaging and therapy. Drug Deliv.

[21] Zhang Z, Hu Z, Gupta J, Krimmel JD, Gerseny HM, Berg AF (2012). Intravenous administration of adenoviruses targeting transforming growth factor beta signaling inhibits established bone metastases in 4T1 mouse mammary tumor model in an immunocompetent syngeneic host. Cancer Gene Ther.

[22] Seth P, Wang ZG, Pister A, Zafar MB, Kim S, Guise T (2006). Development of oncolytic adenovirus armed with a fusion of soluble transforming growth factor-beta receptor II and human immunoglobulin Fc for breast cancer therapy. Hum Gene Ther.

[23] Hu Z, Gerseny H, Zhang Z, Chen YJ, Berg A, Zhang Z (2011). Oncolytic adenovirus expressing soluble TGFβ receptor II-Fc-mediated inhibition of established bone metastases: a safe and effective systemic therapeutic approach for breast cancer. Mol Ther.

[24] Hu Z, Gupta J, Zhang Z, Gerseny H, Berg A, Chen YJ (2012). Systemic delivery of oncolytic adenoviruses targeting transforming growth factor-β inhibits established bone metastasis in a prostate cancer mouse model. Hum Gene Ther.

[25] Xu W, Neill T, Yang Y, Hu Z, Cleveland E, Wu Y (2015). The systemic delivery of an oncolytic adenovirus expressing decorin inhibits bone metastasis in a mouse model of human prostate cancer. Gene Ther.

[26] Yang Y, Xu W, Neill T, Hu Z, Wang CH, Xiao X (2015). Systemic delivery of an oncolytic adenovirus expressing decorin for the treatment of breast cancer bone metastases. Hum Gene Ther.

[27] Dai S, Lv Y, Xu W, Yang Y, Liu C, Dong X (2020). Oncolytic adenovirus encoding LIGHT (TNFSF14) inhibits tumor growth via activating anti-tumor immune responses in 4T1 mouse mammary tumor model in immune-competent syngeneic mice. Cancer Gene Ther.

[28] Zhao H, Wang H, Kong F, Xu W, Wang T, Xiao F (2019). Oncolytic Adenovirus rAd.DCN Inhibits Breast Tumor Growth and Lung Metastasis in an Immune-Competent Orthotopic Xenograft Model. Hum Gene Ther.

[29] Kumar A, Taghi Khani A, Sanchez Ortiz A, Swaminathan S (2022). GM-CSF: A Double-Edged Sword in Cancer Immunotherapy. Front Immunol.

[30] Dahmani A, Delisle J-S (2018). TGF-β in T Cell Biology: Implications for Cancer Immunotherapy. Cancers.

[31] Han J, Khatwani N, Searles TG, Turk MJ, Angeles CV (2020). Memory CD8+ T cell responses to cancer. Semin Immunol.

[32] Kim CW, Kim KD, Lee HK (2021). The role of dendritic cells in tumor microenvironments and their uses as therapeutic targets. BMB Rep.

[33] Veglia F, Sanseviero E, Gabrilovich DI (2021). Myeloid-derived suppressor cells in the era of increasing myeloid cell diversity. Nat Rev Immunol.

[34] Boutilier AJ, Elsawa SF (2021). Macrophage polarization states in the tumor microenvironment. Int J Mol Sci.

[35] Blanchette P, Teodoro JG (2023). A renaissance for oncolytic adenoviruses?. Viruses.

[36] Geurts V, Kok M (2023). Immunotherapy for metastatic triple negative breast cancer: current paradigm and future approaches. Curr Treat Options Oncol.

[37] Guo ZS, Liu Z, Bartlett DL (2014). Oncolytic immunotherapy: dying the right way is a key to eliciting potent antitumor immunity. Front Oncol.

[38] Syed V (2016). TGF-β signaling in cancer. J Cell Biochem.

[39] Lee EHJ, Murad JP, Christian L, Gibson J, Yamaguchi Y, Cullen C (2023). Antigen-dependent IL-12 signaling in CAR T cells promotes regional to systemic disease targeting. Nat Commun.

[40] Larson RC, Kann MC, Bailey SR, Haradhvala N, Llopis PM, Bouffard A (2022). CAR T cell killing requires the IFNγR pathway in solid but not liquid tumours. Nature.

[41] Derakhshan F, Reis-Filho JS (2022). Pathogenesis of Triple-negative breast cancer. Annu Rev Pathol.

[42] Schrörs B, Boegel S, Albrecht C, Bukur T, Bukur V, Holtsträter C (2020). Multi-Omics characterization of the 4T1 Murine mammary gland tumor model. Front Oncol.

[43] Jiao S, Subudhi SK, Aparicio A, Ge Z, Guan B, Miura Y (2019). Differences in Tumor Microenvironment Dictate T Helper Lineage Polarization and Response to Immune Checkpoint Therapy. Cell.

[44] Schreiber K, Karrison TG, Wolf SP, Kiyotani K, Steiner M, Littmann ER (2020). Impact of TCR Diversity on the Development of Transplanted or Chemically Induced Tumors. Cancer Immunol Res.

[45] Cowell LG (2020). The Diagnostic, Prognostic, and Therapeutic Potential of Adaptive Immune Receptor Repertoire Profiling in Cancer. Cancer Res.

