# Supplementary material for: The safety and efficacy of systemic delivery of a new liver-de-targeted TGFβ signaling inhibiting adenovirus in an immunocompetent triple negative mouse mammary tumor model
Source: Cancer Gene Ther. 2024 Jan 24;31(4):574–85. doi: 10.1038/s41417-024-00735-1 (PMC11016465; doi:10.1038/s41417-024-00735-1)

**The Safety and Efficacy of Systemic Delivery of a New Liver-de-targeted TGFβ Signaling Inhibiting Adenovirus in an Immunocompetent Triple Negative Mouse Mammary Tumor Model**

Soon Cheon Shin**^1*^**, Renee E. Vickman**^2*^**, Beniamin Filimon**^1^**, Yuefeng Yang**^1,3^**, Zebin Hu**^1,4^**, Kathy A. Mangold**^5,7^**, Bellur S. Prabhakar**^6^**, Hans Schreiber**^7^**, and Weidong Xu**^1✉^**

**^1^**Cancer Gene Therapy Program, Department of Medicine, NorthShore University HealthSystem, an Academic Affiliate of the University of Chicago Pritzker School of Medicine, Evanston, Illinois, USA; **^2^**Center for Personalized Cancer Care, Department of Surgery, NorthShore University HealthSystem, an Academic Affiliate of the University of Chicago Pritzker School of Medicine, Evanston, Illinois, USA; **^3^**Department of Experimental Medical Science and Key Laboratory of Diagnosis and Treatment of Digestive System Tumors of Zhejiang Province, Ningbo, China; **^4^**National Institutes for Food and Drug Control, Beijing, China; **^5^**Department of Pathology and Laboratory Medicine, NorthShore University HealthSystem, Evanston, Illinois, USA; **^6^**Department of Microbiology and Immunology, University of Illinois College of Medicine, Chicago, Illinois, USA; **^7^**Department of Pathology, The University of Chicago, Chicago, Illinois, USA.
*: These authors contributed equally to this work. SUPPLEMENTARY INFORMATION (data are in separated files)

**SUPPLEMENTARY INFORMATION**

SUPPLEMENTARY TABLE 1. Primer efficiency and specificity information

SUPPLEMENTARY TABLE 2. Comparison of the toxicity profiles between mHAdLyp.sT and other tested adenoviruses or untreated group

SUPPLEMENTARY FIGURE. Flow cytometry gating strategy of T cells and myeloid cells. (A) Representative images of gating strategy for identification of specific T cell subpopulations; (B) Representative images of gating strategy for identification of specific myeloid cell subpopulations.

**SUPPLEMENTARY TABLE 1** (The excel file is uploaded separately) **
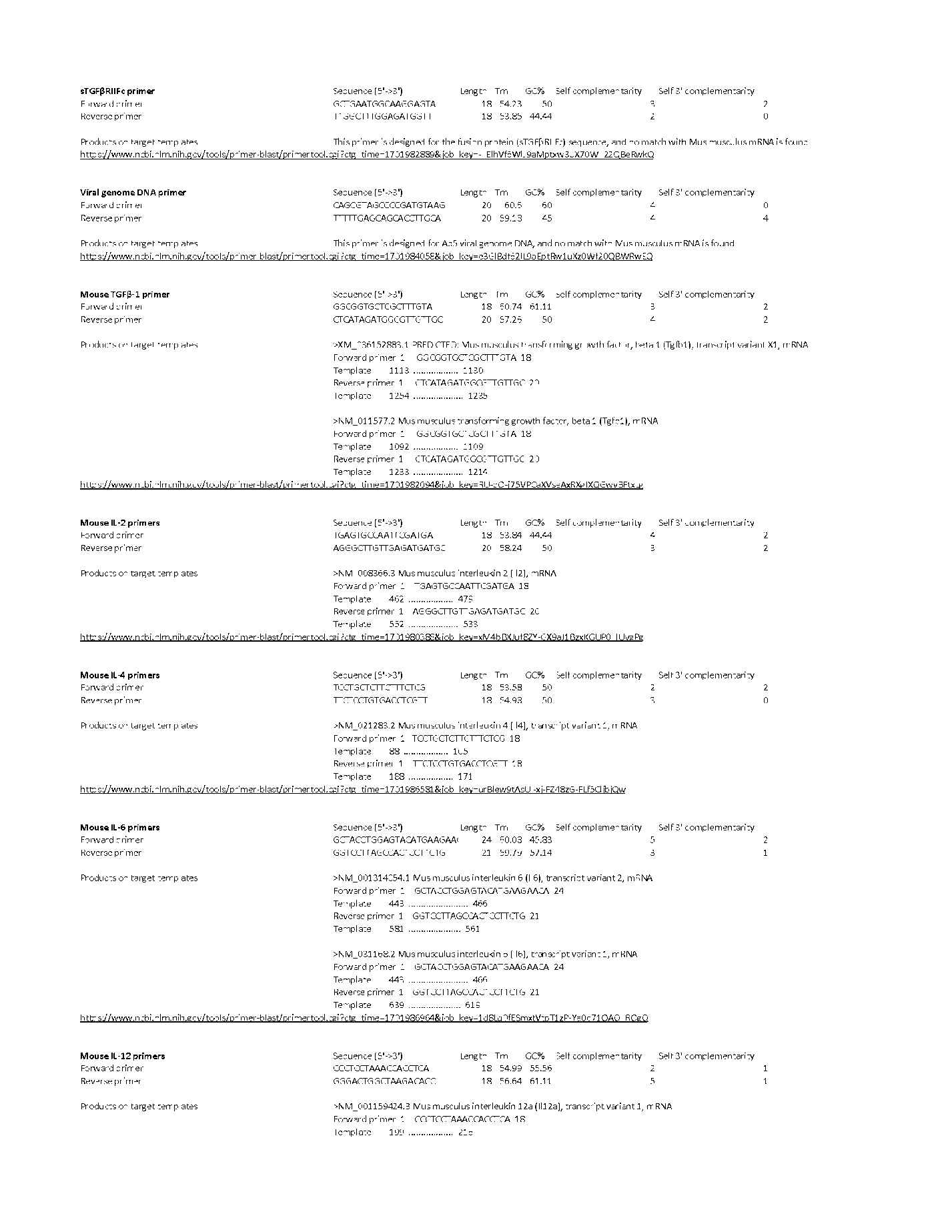

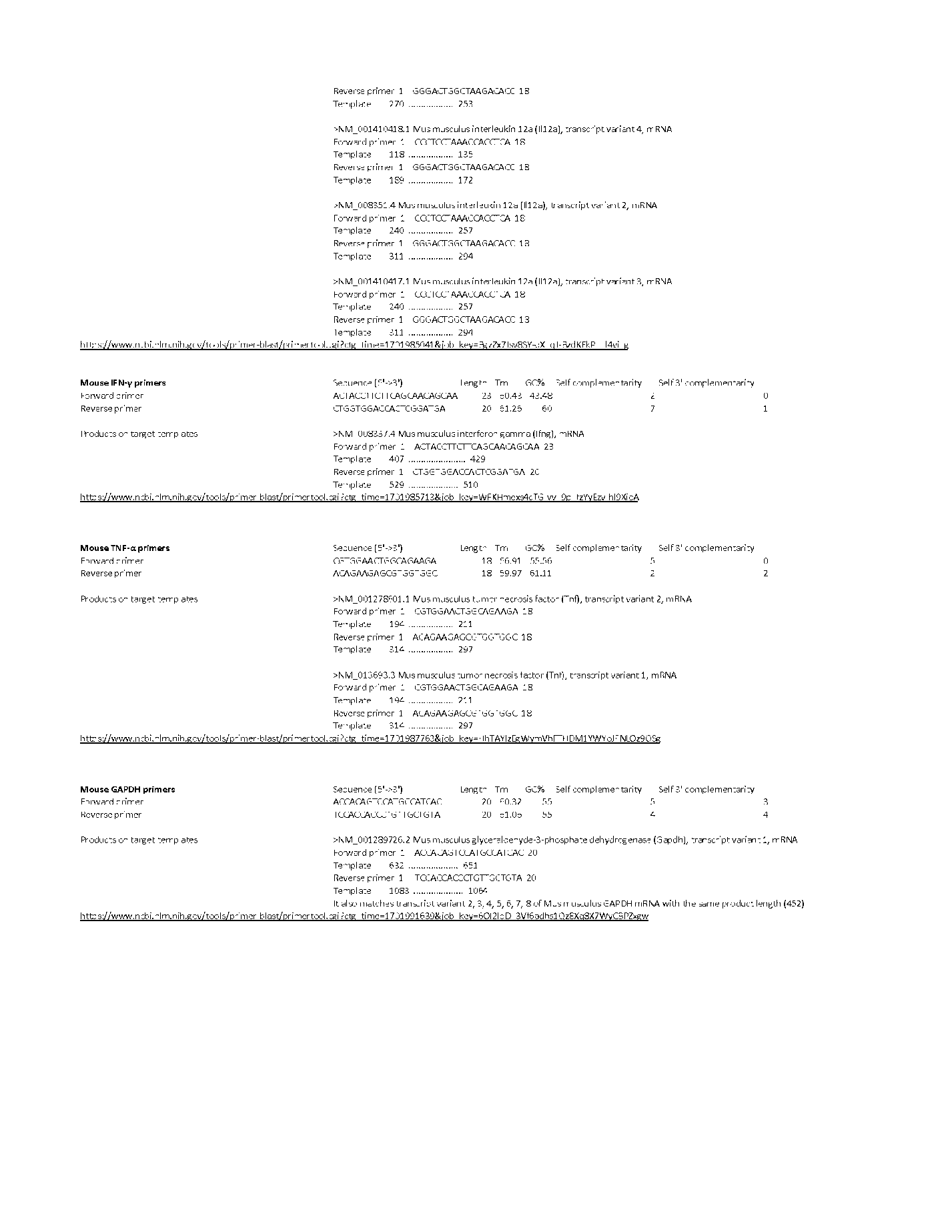
**

**SUPPLEMENTARY TABLE 2** (The excel file is uploaded separately)

|  | **Hepatic and systemic toxicity** | | | **Proinflammatory cytokine responses** | | | | | | |
| --- | --- | --- | --- | --- | --- | --- | --- | --- | --- | --- |
|  | LDH | ALT | AST | IL-4 | IL-6 | IL-10 | IL-2 | TNF-α | IL-12 | IFN-γ |
| **Buffer** |  |  |  |  |  |  |  |  | * | * |
| **Ad(E-).null** |  |  |  |  |  |  |  |  |  |  |
| **Ad.sT** | * | ** |  | ** | * | * | * | ** |  |  |
| **AdLyp.sT** | * |  |  |  | * |  |  | * |  |  |
| **mHAd.sT** |  |  |  |  |  |  |  |  |  |  |
| Significant differences were obtained by comparing to **mHAdLyp.sT** by One-Way ANOVA with Dunn’s multiple comparisons tests (* = p < 0.05, ** = p < 0.01). | | | | | | | | | | |

**SUPPLEMENTARY FIGURE**
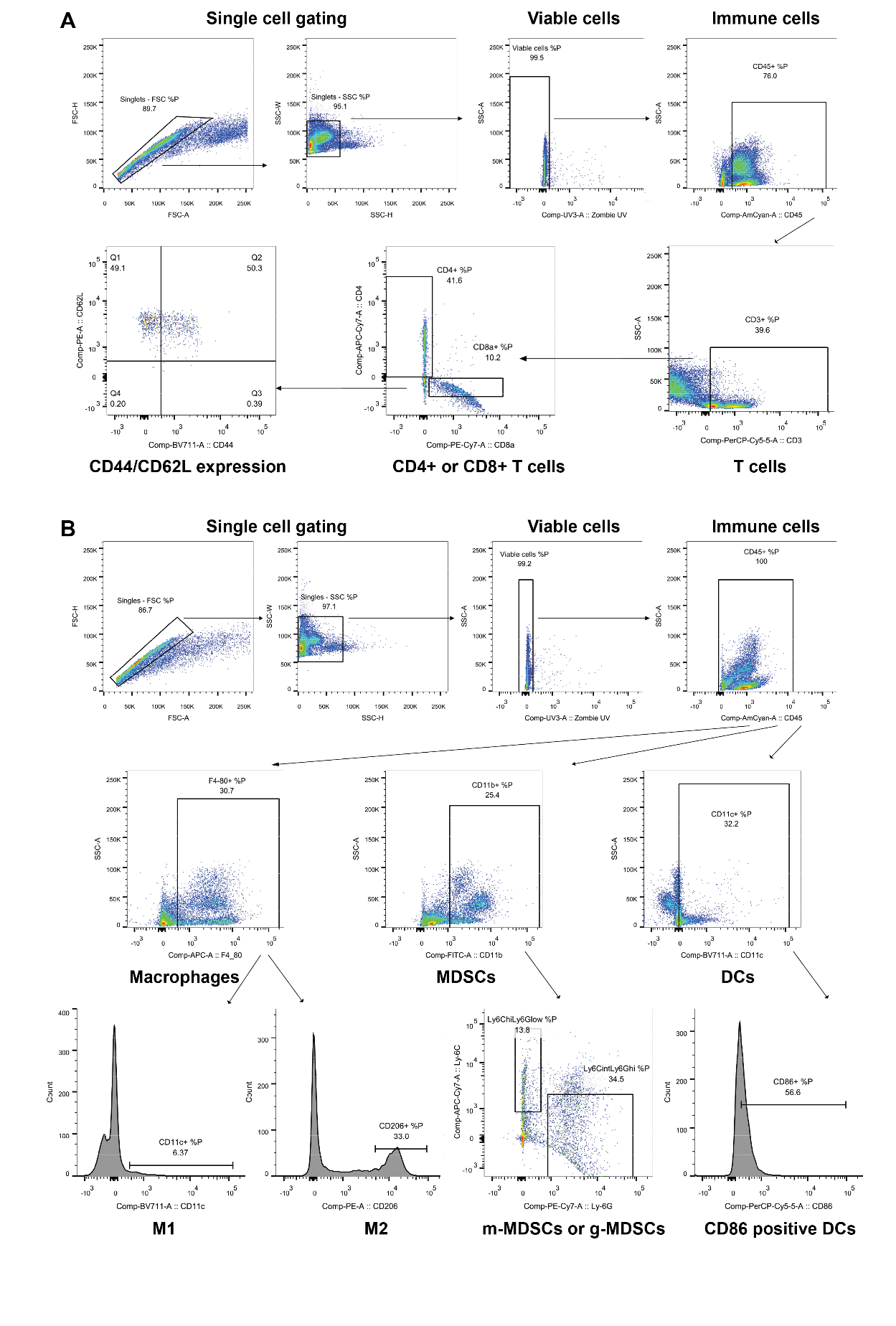

Supplement: Supplementary file 1 — Supplemental material merged [file 41417_2024_735_MOESM1_ESM.docx]
